# Fifty years of HLA-associated type 1 diabetes risk: history, current knowledge, and future directions

**DOI:** 10.3389/fimmu.2024.1457213

**Published:** 2024-09-12

**Authors:** Janelle A. Noble

**Affiliations:** ^1^ Children’s Hospital Oakland Research Institute, Oakland, CA, United States; ^2^ University of California San Francisco, Oakland, CA, United States

**Keywords:** Type 1 diabetes, autoimmunity, HLA, immune polymorphism, disease association, diversity,

## Abstract

More than 50 years have elapsed since the association of human leukocyte antigens (HLA) with type 1 diabetes (T1D) was first reported. Since then, methods for identification of HLA have progressed from cell based to DNA based, and the number of recognized HLA variants has grown from a few to tens of thousands. Current genotyping methodology allows for exact identification of all HLA-encoding genes in an individual’s genome, with statistical analysis methods evolving to digest the enormous amount of data that can be produced at an astonishing rate. The HLA region of the genome has been repeatedly shown to be the most important genetic risk factor for T1D, and the original reported associations have been replicated, refined, and expanded. Even with the remarkable progress through 50 years and over 5,000 reports, a comprehensive understanding of all effects of HLA on T1D remains elusive. This report represents a summary of the field as it evolved and as it stands now, enumerating many past and present challenges, and suggests possible paradigm shifts for moving forward with future studies in hopes of finally understanding all the ways in which HLA influences the pathophysiology of T1D.

## 1 Introduction

The human leukocyte antigen (HLA) region genes are well established to be the strongest contributors to genetic risk for type 1 diabetes (T1D) (1, 2). The association of HLA with T1D was first reported more than 50 years ago (3), closely following a report of “no relation” of HL-A with juvenile diabetes (4). Since then, more than 5,000 reports have been published on the study of HLA-associated T1D risk. Early studies reported data generated from cell-based, serologic assays. With the introduction of polymerase chain reaction (PCR) in the 1980s, DNA-based HLA genotyping methods were developed, first using differential amplification- (SSP: sequence-specific priming) or hybridization- (SSOP: sequence-specific oligonucleotide probes) based methods, progressing to DNA sequencing of amplified targets (SBT: sequence-based typing), and leading to next generation sequencing (NGS) of increasingly larger regions of the HLA genes. The latest improvement in sequencing technology methods is long read sequencing, which was dubbed the “method of the year” for 2022 and can allow sequence reads tens of thousands of bases long (5). This method enables detection of all polymorphisms in exons, introns, and untranslated sequences in any given HLA gene and facilitates direct determination of phase (assignment of sequence reads to a particular chromosome) and direct determination of haplotypes (sets of HLA alleles found *in cis* on one chromosome). The number of HLA loci, the extreme polymorphism of those loci, and the strong linkage disequilibrium (LD: non-random tendency of alleles to be found together) between particular loci, create a complex system where results can easily be confounded and misinterpreted. Other polymorphic immune system genes, such as killer-cell immunoglobulin-like receptors (KIRs), found on NK cells, and leukocyte immunoglobulin-like receptors (LILR), primarily found on myeloid antigen-presenting cells, are less well understood, although some have been implicated in T1D risk. Even with more than 50 years of study, the basis of HLA-associated T1D risk is not completely elucidated. The recent Food and Drug Administration approval of teplizumab as a treatment to delay beta-cell loss increases the urgency for early detection of T1D (6–9). Identification of individuals at high T1D risk is important for optimal disease monitoring and patient stratification for use of teplizumab and other, future treatment and prevention strategies. This report is intended to provide historical perspective on the association of HLA with T1D, summarize the current state of the field, and provide suggestions for moving the field forward with the goal that, one day, the complexity of HLA-associated T1D risk will finally be fully understood and will play a key role in accurate diagnosis, management, precision treatment, and even prevention of the disease.

## 2 The basics of HLA structure and function

### 2.1 *HLA* antigens

Classical HLA antigens are cell-surface molecules that include a peptide binding groove in which either endogenous (for HLA class I) or exogenous (for HLA class II) peptides bind and are presented to the T-cell receptor, creating the “tri-molecular complex.” Signaling through this complex initiates the T cell response to the antigenic peptide, which can result in anergy, regulatory function, or effector function. Both HLA class I and class II proteins have a similar external structure, with the peptide-binding groove formed by amino acid residues in two immunoglobulin-like domains; however, class II antigens are heterodimeric proteins comprised of separately encoded alpha and beta polypeptides, while class I antigens are comprised of a single polypeptide chain that forms a complex with the relatively invariant β2-microglobulin molecule (2).

The genes encoding classical HLA (class I A, B, and C and class II DR, DQ, and DP) are the most polymorphic known in the human genome. As of December 2023, the number of alleles assigned in the IPD-IMGT/HLA allele database for classical HLA loci was 38,008 (10). Early, cell-based genotyping systems only distinguished a small number of categories for each locus. Rapid increases in identification of individual alleles began with the advent of DNA-based typing methods in the early 1990s. Even more rapid expansion of the catalog of named alleles was fueled by the development of NGS-based HLA genotyping methods around 2010, and, more recently, advances in long-read sequencing technology have led to the ability to sequence through and phase the entire ~4 Mb HLA region. Not surprisingly, most polymorphic sites in HLA molecules are located in the exons encoding the peptide-binding groove, creating the basis for presentation of a large repertoire of peptides for each of the classical HLA antigens. Exons encoding the peptide-binding groove (exon 2 in class II and exons 2 and 3 in class I) were the first to be targeted in molecular genotyping methods. Current methods can determine the entire sequence of an HLA gene, including exons, introns, and untranslated sequences, allowing exact identification of the alleles in a genotype. In addition, high-depth coverage and long read sequencing allow phase determination and assignment of individual alleles to haplotypes on each chromosome (11).

### 2.2 Nomenclature

Nomenclature for HLA has evolved over many years. The current standard includes the locus name, followed by an asterisk separator, followed by up to four numeric fields with colon separators, and, in some cases, by a letter (N, L, S, C, A, or Q) indicating expression status (12). For most loci, the first field describes the allele group, generally related to defined serologic specificity, the second field represents the individual allele within the group as defined by differences in the encoded amino acids, the third field reflects silent changes in the codons, and the fourth field reflects variation in intron and untranslated sequences. The *DPB1* locus differs from others in that most alleles have been discovered since DNA-based genotyping was implemented. For nearly all *DPB1* alleles, the first field simply represents the order in which the alleles were discovered, rather than a particular, serologically defined allele group, and the second field is almost exclusively “01.” A review of the history of HLA nomenclature was recently published (13).

Given the cumbersome nature of the individual allele designations, for example, HLA-A*01:01:01:01N, many reports use abbreviated definitions for particular alleles. For example, the allele DRB1*03:01:01:01 is sometimes referred to as simply “DR3” but can be referred to using up to four fields of resolution. In some reports, the term “DR3” may refer to the individual allele at the *DRB1* locus or may refer to haplotypes that carry the DRB1*03:01:01:01 allele. Thus, interpretation of data from association studies must be done with careful consideration of the genotyping methodology, the resolution, and the authors’ preferences in reporting nomenclature. Despite efforts to standardize reporting information for HLA genotyping, results for individual studies still vary in the nomenclature used (14).

**Figure 1:**
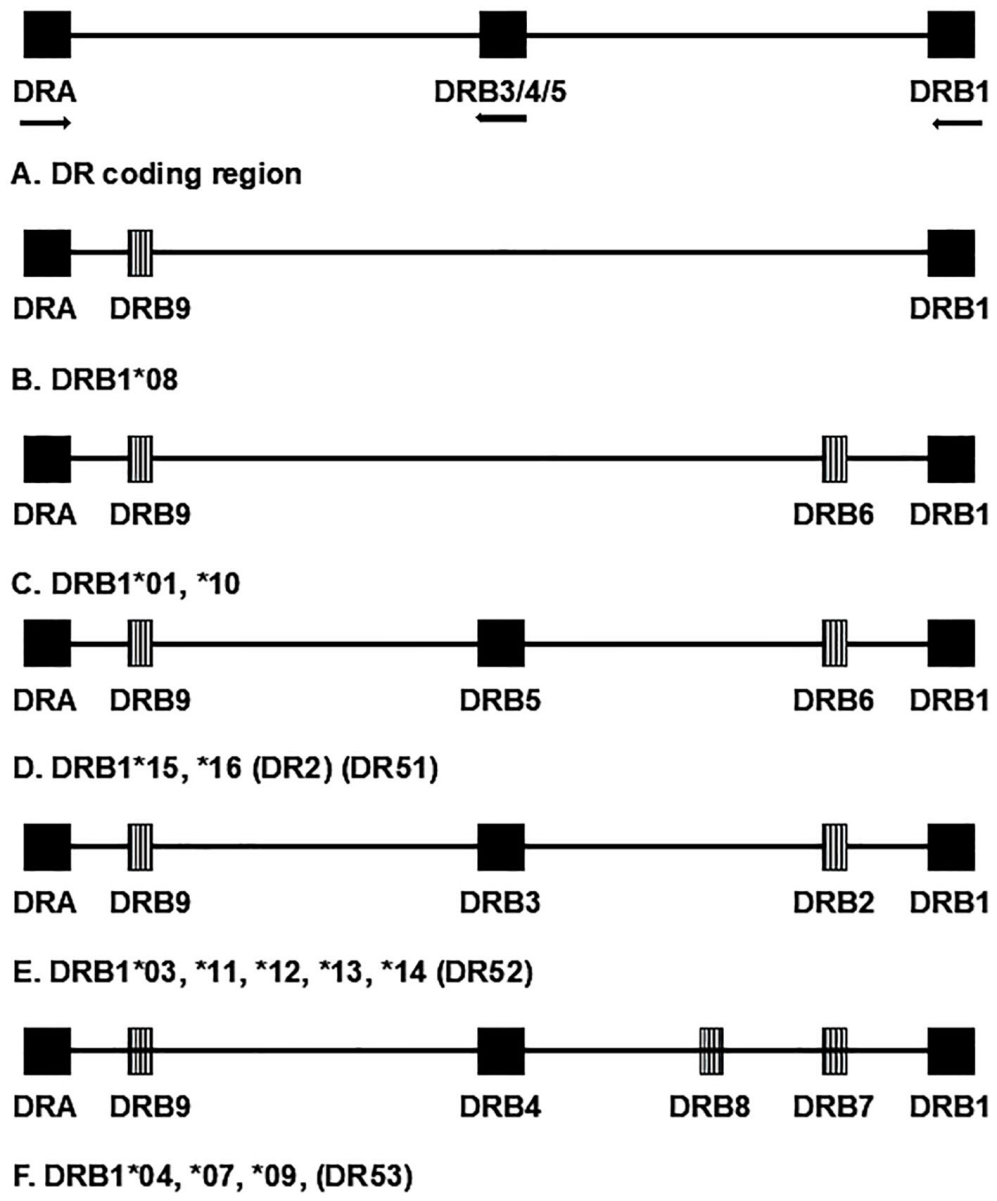
Schematic representation of chromosomal region encoding HLA-DR antigens; not to scale. Although they are separate loci, *DRB3*, *DRB4*, and *DRB5* are separate loci but are represented as a single locus for convenience. Expressed loci are represented by solid blocks. Striped blocks represent pseudogenes. Arrows designate direction of transcription for expressed genes. Chromosome groups are designated by the first-field nomenclature, for example, DRB1*01, which represents the corresponding serologic group. Alternate, historical name categories for the groups are given in parentheses.

Class I antigens (HLA-A, -B, and -C) are formed by a single polypeptide chain (combined with β2-microglobulin); their designations refer to a single locus but can vary from simple designations that only reflect specificity at the serologic level (e.g., “A1”) to complete whole gene (four-field) specificity with expression data (e.g., A*01:01:01:01N). Class II antigens (HLA-DR, -DQ, and -DP) are heterodimers composed of the products of two genes (“A” and “B,” encoding the α and β polypeptide chains, respectively). For genes encoding DR, nearly all the polymorphism is found in the *DRB* genes, with the variability in the *DRA1* gene so low that the locus is usually not genotyped. Thus, nomenclature for DR-encoding loci generally consists simply of a description of the *DRB* allele. All HLA regions contain a *DRB1* gene; however, other *DRB* genes, both the expressed *DRB3*, *DRB4*, and *DRB5* loci and the pseudogenes *DRB2*, *DRB6*, *DRB7*, *DRB8*, and *DRB9*, are present on some, but not all, chromosomes (**Figure 1**). They are found in distinct LD patterns with particular *DRB1* alleles. Each chromosome carries a maximum of one secondary, expressed *DRB* locus (i.e., *DRB3*, *DRB4*, *DRB5*) (see section 3.7). Thus, although they are separate loci, *DRB3*, *DRB4*, and *DRB5* can be analyzed as if they were alleles of one locus.

In contrast, for genes encoding DQ, both the *DQA1* and *DQB1* loci are polymorphic, and both must be genotyped to fully describe the DQ antigen. One way to do this is to write out the full *DQA1-DQB1* haplotype (*e.g.*, DQA1*05:01~DQB1*02:01); however, for brevity, many reports use abbreviations to represent the haplotype. In fact, these abbreviations describe the heterodimeric antigen formed by the combination of polypeptide products of the *DQA1* and *DQB1* loci. Common DQ abbreviations relevant to T1D risk include DQ2.5 (DQA1*05:01~DQB1*02:01), DQ8 (DQA1*03:01~DQB1*03:02), DQ7 (DQA1*05:01~DQB1*02:01), DQ6.2 (DQA1*01:02~DQB1*06:02), and DQ9.3 (DQA1*03:02~DQB1*03:03).

For DP, as for DQ, genes encoding both chains of the antigen are polymorphic. Consistent with other class II antigens, the *DPB1* gene is far more polymorphic than is the *DPA1* gene, with 2,486 and 639 reported alleles, respectively (10). Until recently, *DPA1* was rarely genotyped, and DP data, when available, were nearly always reported in the same way as DR data, simply by using the allele designation for the *DPB1* gene. As genotyping technology evolves, more reagents are becoming available for genotyping *DPA1*, particularly since many assay systems are now multiplexed. Therefore, the proportion of reported *DPA1*~*DPB1* haplotype data, compared to *DPB1* data alone, is increasing. While some *DPB1* alleles are seen in haplotype with a single *DPA1* allele, others, like DPB1*02:01, can be seen *in cis* with one of multiple *DPA1* alleles (15). Data reports for DP association do not utilize abbreviations analogous to those used for DQ association. Interpretation of DP genotyping results requires information about whether the data presented represent only the *DPB1*-encoded portion of the antigen (β chain) or both the *DPA1* and DPB1-encoded portions (α and β chains). Since both the *alpha* and the beta chains contribute to the shape of the antigen-binding groove, identification of both chains is important to fully understand the structure of the resulting DP antigen. Given the fact that both *DPA1* and a *DPB1* products contribute to the peptide binding groove of the DP antigen, and given the discovery that some DP antigens can serve as ligands for NK cell proteins and influence innate immunity, identification of both the *DPA1* and *DPB1* loci in an individual is becoming increasingly important (16, 17).

### 2.3 Resolution

Generation and analysis of data at the highest resolution possible (four-field) for any study involving HLA genotyping may seem optimal, but highest resolution is not always the best basis for an association study. Increased resolution of a genotyping study generally involves increased cost. The perfect disease association study would sample every individual in the entire global population and sequence every genome in its entirety, providing a complete data set that could be stratified by any genetic criteria desired, for example, using Ancestry Informative Markers (AIMs), before application of any association analysis method. Clearly, such a study is neither practical nor even possible. In the absence of this ideal, each study design must consider the level of resolution necessary to best address the hypothesis being tested, taking into account the resources available for the study. Most studies of HLA and disease association report data at a resolution of two fields (previously referred to as “4-digit” resolution), because the first two fields contain all the information defining the amino acids encoded for the protein. Changes in the third or fourth field do not affect which amino acids are included in a given allele. In rare cases, information found in third or fourth fields might be relevant to gene expression, through potential mechanisms such as altered codon usage, changes in splice donor or acceptor sequences, or presence of an enhancer. On a practical level, however, third and fourth field information adds little to a disease association study. The primary function of HLA molecules is to present peptides; thus, two molecules that contain coding sequence for the same string of amino acid residues are expected to have the same structure and bind the same repertoire of peptides. If the coding sequence for those two antigens differs by silent changes in codons or untranslated sequences, considering them as different alleles in an association analysis could effectively decrease the statistical power of the study and mask association effects. Since three- or four-field resolution data can be collapsed to two-field resolution, generating data at the highest level of resolution within the scope of the study is desirable. However, for cases in which generating data at four-field resolution is not possible or greatly reduces the number of samples that can be tested with the resources available for a project, generating data at the two-field level (coding sequence only) may be the preferred option. Each disease association project is unique, and many studies have been, and continue to be, performed with low- or moderate-resolution output, creating useful data but also creating challenges for comparing or combining data among studies.

Varying resolution represents a significant problem not only for disease association studies but also for combining data in any HLA-related study involving samples that were genotyped at varied levels of resolution. For example, large transplant registries, such as the National Marrow Donor Program (NMDP), have data spanning the evolution of genotyping technology, creating challenges for optimizing matching of potential donors to recipients. One report from the NMDP approached this issue by examination of 6.59 million subjects categorized into 21 race categories, with over 1.2 million European Caucasian subjects, using expectation maximization algorithms to resolve allelic ambiguity and phase haplotypes (18). A similar strategy may be applicable for combining data from a large number of disease association studies. For resolution, decreasing the level is simple. Increasing the level is more complex and must be done by informatics technology. While not as accurate as direct genotyping of all samples, imputation of high-resolution data from low-resolution data can aid in allowing the generation of large and meaningful datasets without additional, and perhaps cost-prohibitive, direct genotyping assays. That said, drawing conclusions from imputed data must always be done with caution and take into account that all data were not experimentally determined. For data with direct clinical consequences, such as matching donor-recipient pairs, imputed data must be confirmed experimentally before performing clinical procedures.

## 3 HLA allele, haplotype, and genotype associations with T1D

### 3.1 Discovery

Early publications of HLA association with T1D reported serologic association of class I antigens “HL-A8” and “W15.” (3, 19, 20) In 1980, Barbosa reported increased frequency of class II antigens then termed “Dw3” and “Dw4” and decreased frequency of “Dw2” as well as increased frequency of the Dw3/Dw4 heterozygous genotype (21). These results still stand, having been replicated and published in thousands of studies. Although the full extent of the effect of HLA on T1D susceptibility remains to be determined, the depth and breadth of the field has expanded enormously over 50 years. T1D risk for HLA has consistently been demonstrated to be strongest for genes encoding HLA-DR and -DQ antigens, with risk clearly apparent for each locus in nearly every published study. The vast majority of T1D risk studies report association data in the form of Odds Ratio (OR), which reflects the frequency of the tested allele, haplotype, or genotype in T1D patients compared to that in controls, and each OR is given with a measure of statistical significance in the form of a probability (*p*) value. An OR of 1.0 denotes no association between the tested parameter and disease; OR greater than 1 denotes potential positive association (predisposition to disease), and OR less than 1 denotes potential negative association (protection from disease). Significance is generally accepted as a *p*-value of 0.05 or lower after appropriate statistical corrections.

### 3.2 DR3, DR4, and the DR3/DR4 genotype

The DRB1*03:01~DQA1*05:01~DQB1*02:01 haplotype, sometimes referred to as “DR3-DQ2.5,” or even simply “DR3,” is positively associated with T1D in nearly every population reported to date, although the strength of the association varies among populations. While the extreme polymorphism of the HLA genes creates a daunting number of potential antigens, in practice, only a small subset of named alleles, and, therefore, antigens encoded by them, is present in any given population. At the two-field level of resolution, 211 DRB1*03 alleles have been reported (10). However, only two of those alleles, DRB1*03:01 and DRB1*03:02 (the latter seen almost exclusively in Black populations) are found at appreciable frequency (≥0.5%), with the remaining alleles too rare to be of use for disease association studies (18). The DQ-encoding haplotype DQA1*05:01~DQB1*02:01 is in very strong LD with DRB1*03:01; thus, stratification on the DRB1*03:01~DQA1*05:01~DQB1*02:01 haplotype allows evaluation of risk for alleles at other HLA loci (e.g., class I, *DPB1*) and non-HLA loci (e.g., *TNFA*) in the region without confounding by heterogeneity of DR- and DQ-encoding loci. Some HLA haplotypes are highly conserved, including the DR3 haplotype sometimes referred to as “B8-DR3,” “A1-B8-DR3,” or “8.1,” which was the second most common haplotype observed in a set of over 6 million samples from the NMDP repository (18). The observation that DR3 haplotypes containing HLA-B*08:01 confers less T1D risk than other DR3 haplotypes, particularly “B18-DR3,” has been consistently reported, beginning as early as 1988 (22–26). Other extended “DR3” haplotypes have been seen in varying populations. For example, A2-B8-DR3, A2-B50-DR3, A33-B58-DR3, A24-B8-DR3, and A26-B8-DR3 are all T1D risk haplotypes in the Indian population, while A33-B17-DR3 was suggested as a risk haplotype in Chinese (27, 28).

Additional class II susceptibility is attributable to “DR4,” which denotes DRB1*04:xx alleles, or haplotypes carrying them. DR4-associated T1D risk is more complex than that of DR3, because DR4 haplotypes are far more varied than DR3. To date, 375 DRB1*04 alleles have been named (10). At least nine of those are reported to be at a frequency ≥1% in at least one population (18). A hierarchy of risk exists for DRB1*04:xx alleles, with DRB1*04:05 representing very high risk, followed by others, including DRB1*04:01, DRB1*04:02, and progressing to DRB1*04:03, which has no risk, but is actually protective, for T1D (2). In addition, the DQ-encoding haplotypes that are found coupled to these *DRB1* alleles can vary. In particular, DQA1*03:01~DQB1*03:02 (DQ8) is well-established to be a T1D susceptibility haplotype, while DQA1*03:01~DQB1*03:01 (DQ7) is generally T1D protective, suggesting that T1D risk is more dependent on DQ than on DR (29, 30). On the other hand, the haplotype DRB1*04:01~DQA1*03:01~DQB1*03:02 is an established T1D risk haplotype, while DRB1*04:03~DQA1*03:01~DQB1*03:02 is T1D protective, suggesting that the T1D risk is attributable to DR and not DQ. This conundrum creates a need for large and diverse data sets to evaluate the effects of individual alleles and haplotypes. In some populations, such as those of European descent, 90%–95% of T1D patients carry either DR3 or DR4 on one chromosome. Due to the increasing availability of large sample sets, studies of the subset of patients who carry neither have become feasible to help elucidate predictors for T1D in individuals without the highest risk (31).

**Figure 2:**
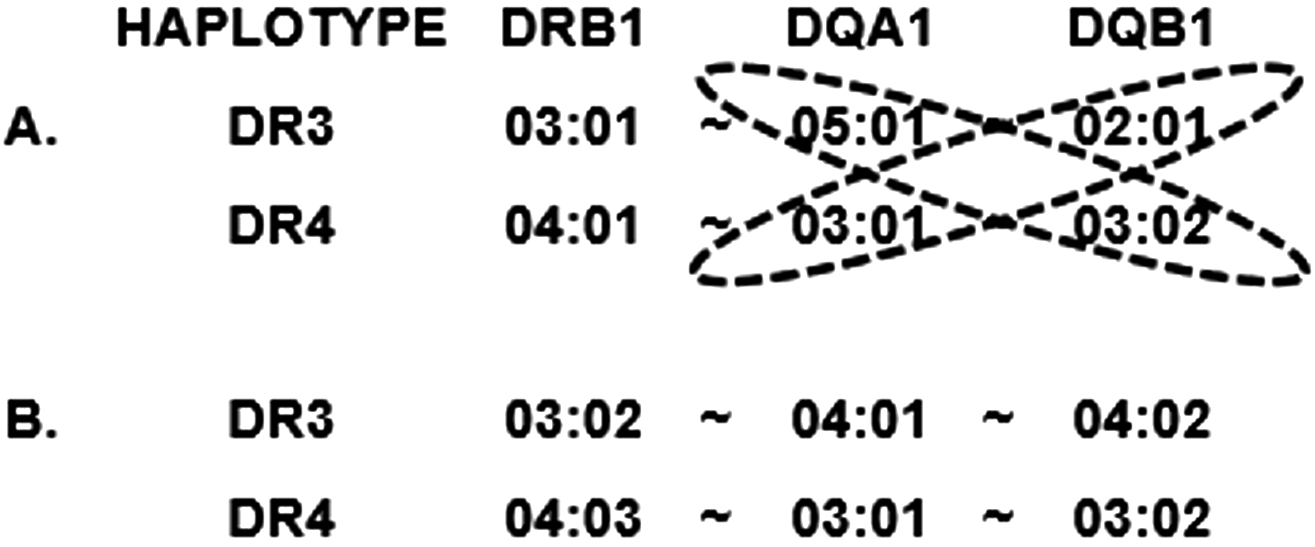
Representation of individual haplotypes that can be found in a “DR3/DR4” heterozygous individual. **(A)** Typical DR3/DR4 genotype for a high-risk individual of European descent. Potential allele pairs that could produce trans-encoded DQ molecules are circled. **(B)** Potential DR3/DR4 haplotype from mixed-population individual with one Asian and one Black parent, composed of two T1D-*protective* haplotypes.

In addition to risk provided by individual HLA alleles and haplotypes, particular HLA genotypes are known to confer risk that is not explained by the additive effects of the alleles or haplotypes that are included in the genotype. The most well-known example of this is the very high risk conferred by the heterozygous “DR3/DR4” genotype, in which one chromosome carries the DR3 haplotype and the other carries a high-risk DR4 haplotype (excluding those containing DRB1*04:03 or DQA1*03:01~DQB1*03:01). Augmented risk for the DR3/DR4 genotype was reported as early as 1980, and the genotype can be seen in up to 45% of patients in some studies (21, 30). In fact, the risk for the DR3/DR4 genotype is so strong that many studies stratify by those patients and analyze them as a separate group from the remaining patients (32–35). For decades, a comprehensive explanation for the more than additive risk of the DR3/DR4 genotype has remained elusive; however, a leading hypothesis involves DQ heterodimeric antigens encoded *in trans* (2). **Figure 2A** depicts the two DR-DQ haplotypes that are commonly found in a high-T1D risk individual of European descent and illustrates the four combinations of *DQA1* and *DQB1* loci whose products could theoretically combine to create a DQ antigen. The combination of DQA1*03:01 and DQB1*02:01, depicted here *in trans*, is frequently seen encoded *in cis* and has been reported to be associated with increased T1D risk (36). Although the combination of DQA1*05:01 and DQB1*03:02 has not been described encoded *in cis*, one report provides functional data to support the genetics-based hypothesis that an antigen formed from the products of those two alleles may increase T1D risk (37). Differences were demonstrated in antigenic peptide repertoire binding profiles for the traditional *cis*-encoded “DQ8” (DQA1*03:01~DQB1*03:02 = “DQ8cis”) antigen and for the *trans*-encoded antigen, (DQA1*05:01~DQB1*03:02 = “DQ8trans”), that are consistent with higher T1D risk for DQ8trans than for DQ8cis. The DR3/DR4 heterozygous genotype seen in **Figure 2A** allows the expression of DQA1*05:01 and DQB1*03:02 in the same individual, creating a unique opportunity for formation of a particularly high-risk DQ molecule.

### 3.3 Other DR and DQ T1D risk alleles, haplotypes, and genotypes

While DR3, DR4, and the DR3/DR4 genotype are the most commonly recognized HLA risk factors for T1D, many other alleles, haplotypes, and genotypes have been reported in 50 years of study. Thomson et al. used a method termed Relative Predispositional Effects (RPE) to look for additional T1D risk alleles beyond DR3 and DR4 and found evidence for risk from DR8 and DR1 (38, 39). Perhaps the most comprehensive study of HLA and T1D risk was the Type 1 Diabetes Genetics Consortium (T1DGC) (40, 41). Notably, HLA association with T1D was not originally intended as a primary outcome for the T1DGC; the purpose of the international study was to discover all the T1D susceptibility loci other than the classical HLA loci, including both immune system and non-immune system genes. However, because of the extremely strong T1D association with HLA, proper evaluation of all genetics-based T1D association studies should include HLA context. This is especially true for loci in the same chromosomal region as the HLA loci (chromosome 6p), including genes in the class III region. As part of the T1DGC effort, over 14,000 individuals, from multiple populations, were genotyped for all classical HLA loci (except *DRB3*, *DRB4*, and *DRB5*) at two-field level with exon-based resolution using PCR-Sequence-Specific Oligonucleotide Probe (PCR-SSOP) technology. Data for DR- and DQ-encoding genes, DP-encoding genes, and HLA class I-encoding genes were analyzed and published (15, 29, 42). **Table 1** shows the most common predisposing and protective DRB1~DQA1~DQB1 haplotypes for T1DGC.

**Table 1:** Highly-significant DR-DQ encoding haplotypes for subjects of European descent resulting from the Type 1 Diabetes Genetics Consortium (T1DGC) study (29).

Haplotype	*DRB1~DQA1~DQB1*	Odds ratio	*P-*value
*predisposing*			
**DR3**	**03:01~05:01~02:01**	**3.64**	**2 × 10^−22^**
**DR4**	**04:05~03:01~03:02**	**11.37**	**4 × 10^−5^**
**DR4**	**04:01~03:01~03:02**	**8.39**	**6 × 10^−36^**
**DR4**	**04:02~03:01~03:02**	**3.63**	**3 × 10^−4^**
*protective*			
**DR2**	**15:01~01:02~06:02**	**0.03**	**2 × 10^−29^**
**DR6**	**14:01~01:01~05:03**	**0.02**	**1 × 10^−6^**
**DR7**	**07:01~02:01~03:03**	**0.02**	**3 × 10^−4^**
**DR7**	**07:01~02:01~02:01**	**0.32**	**2 × 10^−9^**
**DR4**	**04:03~03:01~03:02**	**0.27**	**0.017**

Bold values are statistically significant.

Most studies of HLA and T1D risk have been performed on individuals of European descent, not only due to the high prevalence of T1D in that group but also due to the relative ease of recruiting subjects compared to other races and ethnicities. Lack of diversity in published studies can create challenges for thorough understanding of HLA associations with T1D and is described more fully in section 4.1 below. HLA allele frequencies vary enormously among populations as do the combinations of those alleles into haplotypes. Studies of subjects not of European descent are critical for comprehensive understanding of HLA-associated T1D risk. An illustrative example can be seen for DR9. The frequency of DRB1*09:01 is high (up to ~15%) in Asian populations, including Japanese, Chinese, and Vietnamese but very low (~1%) in European populations (18). In Europeans, an association of DRB1*09:01 with T1D would require a very large study to be observed; however, association with DR9, and particularly with the DR3/DR9 genotype, was reported many decades ago in Asian populations (27, 43–45). Another genotype that is relatively uncommon, DR4/DR8, has been shown to be a risk factor for T1D, both in individuals of European descent and in Japanese (46). The increasing admixture among populations can further confound data interpretation. For example, the DR3 haplotype that is exceedingly rare in Europeans but common in Africans (DRB1*03:02~DQA1*04:01~DQB1*04:02) (see section 3.2) is actually protective, rather than predisposing, for T1D (36). Similarly, the DR4 haplotype common in Asians (DRB1*04:03~DQA1*03:01~DQB1*03:02) is protective, rather than predisposing, for T1D. Finding both of these haplotypes in a single individual is unlikely in non-admixed populations but could easily happen in an admixed population, where a child might have one Asian and one Black parent. In that case, a low-resolution genotyping could produce a result of a called DR3/DR4 genotype, leading to an assumption of high T1D risk, in an individual whose HLA genotype is highly protective. This hypothetical example (depicted in **Figure 2B**) highlights the fact that both resolution and population context are important in evaluating data from HLA and T1D association studies.

### 3.4 T1D protection from DR and DQ-encoding alleles

HLA not only confers risk for T1D but also strong protection. The 1980 publication that implicated class II, rather than class I, HLA in T1D risk also reported a decrease in the “Dw2” antigen, which is sometimes termed “DR2” and includes the DRB1*15 and DRB1*16 allele groups (21). In studies of individuals of European descent, the haplotype DRB1*15:01~DQA1*01:02~DQB1*06:02 is, by far, the most frequent DR2-encoding haplotype observed and is consistently associated with T1D protection (29, 30, 39). The closely related haplotype DRB1*15:03~DQA1*01:02~DQB1*06:02 (African-specific) is also highly protective for T1D (36). Importantly, however, the alleles included in DR2-encoding haplotypes and their frequencies vary widely among populations and not all DR2-endocding haplotypes are highly protective (18, 36, 47). DQB1 genes that encode aspartic acid at position 57 are usually protective for T1D, although not universally (48–50). DR2-based T1D protection appears dominant, although the mechanism for the protection is, as yet, unknown. One leading hypothesis is “epitope stealing,” that is, competition for binding of antigenic peptides (51, 52). Another is related to the high stability of the DP antigen encoded by DQA1*01:02~DQB1*06:02 (53–56). DR2 appears to be protective throughout the course of disease, from reducing risk of developing autoantibodies to reducing risk of progression to overt diabetes in individuals with antibody positivity (57).

Other class II haplotypes appear protective for T1D, including DRB1*14:01~DQA1*01:01~DQB1*05:03 and DRB1*07:01~DQA1*02:01~DQB1*02:02 (29). DR7-based T1D protection was observed in early studies of European-descent individuals but not as frequently in individuals of African descent (30, 58). The African version of the DR7 haplotype is DRB1*07:01~DQA1*03:01~DQB1*02:02, differing from the European version only at *DQA1*. That difference modifies the risk for the haplotype from protective (OR = 0.34) to predisposing (OR = 3.96), demonstrating the need for genotyping a more than a single locus to determine T1D risk (59). Other alleles and haplotypes can be T1D protective as well, but with less uniformity of observed risk effects. Alleles in the DRB1*11 and DRB1*13 groups can be found encoded *in cis* with many different *DQA1-DBB1* haplotypes. T1D risk effects from DR11 and DR13 haplotypes vary widely within and among populations.

### 3.5 DPB1

HLA-DP is the remaining of the three class II classical HLA antigens. Like DR and DQ, DP is encoded by polymorphic genes and presents peptide antigens to T-cell receptors, making it a logical candidate to convey T1D risk. Association of the *DPB1* locus with T1D has been examined in many studies and in many populations, although some reports have suggested no association of DP with T1D (15, 26, 34, 60–65). The association of *DPB1* with T1D tends to be less strong and less consistent than that of the DR- and DQ-encoding loci (15). Perhaps the most consistently observed T1D association for *DPB1* and T1D is a protective effect for DPB1*04:02, particularly notable on DR3 haplotypes; however, a recent study from Mali showed a very highly *predisposing* effect for DPB1*04:02 (OR = 12.73) in that population, which, upon closer scrutiny, appeared to be attributable to the presence of DPB1*04:02 on a highly T1D-predisposing extended HLA haplotype rather than to an effect of the allele itself (30, 34, 60, 62, 66). The mechanism(s) for the influence of *DPB1* on T1D remain to be fully elucidated. Possibilities include direct effects of peptide binding for adaptive immunity, LD with other risk loci, or a novel mechanism that may involve the innate immune system (see section 5.2 below).

### 3.6 HLA class I A, B, and C

Original reports for associations of HLA with T1D were for class I serotypes; however, those associations were later shown to be attributable to HLA class II loci in LD with loci encoding those serotypes (3, 19–21). T1D is characterized by an adaptive immune response that leads to destruction of pancreatic beta cells. T-cell killing involves class I HLA molecules; thus, a reasonable assumption is that HLA class I genes might confer susceptibility to T1D, particularly affecting beta cell destruction and disease progression. To be performed properly, HLA class I analyses must account for LD with class II DR- and DQ-encoding genes. Many studies have been published examining class I associated T1D risk (HLA-A, -B, and -C) (26, 33, 42, 67–69). A*24, specifically, A*24:02, is the most commonly reported T1D risk allele for *HLA-A* (33, 42, 69, 70). A*24 has been associated with low residual beta-cell function, rapidly progressing disease, and poor outcome for islet allografts (71–75). HLA-B*39:06 is the most commonly and consistently T1D-associated allele reported for the *HLA-B* locus and, like A*24:02, is reported to drive disease progression, including in a humanized mouse model (42, 67–70, 75, 76). *HLA-C* is the least commonly studied of the class I loci for T1D risk, and no allele has shown consistent associations among studies (42, 67, 77). In fact, in a recent study of HLA and T1D in Malian populations, The *HLA-C* and *DPA1* loci were the only classical HLA loci not to reach statistically significant association at the locus level (66). Observed associations of particular *HLA-C* alleles with T1D can frequently be attributed to LD with class II or with *HLA-B* alleles. The lack of *HLA-C* alleles demonstrating consistent T1D risk effects among populations leads to the notion that the influence of *HLA-C* on T1D risk may be attributable to a mechanism different from the traditional presentation of antigen to T cells through the tri-molecular complex. Other mechanisms could include the role of the *HLA-C* antigen as a ligand for Killer cell Immunoglobulin-like Receptors (KIR, see section 5.2) or a different, as yet unidentified, mechanism unrelated to traditional antigen presentation.

### 3.7 DRB3, DRB4, and DRB5

The HLA *DRB3*, *DRB4*, and *DRB5* genes, like *DRB1*, encode DRβ polypeptides that can form heterodimers with the product of the *DRA* gene to produce functional DR antigens. Unlike *DRB1*, these loci are not found on every copy of chromosome 6 but are present, in predictable LD patterns, in HLA regions with particular *DRB1* loci. Each chromosome has a maximum of one secondary, coding *DRB* locus, shown schematically in **Figure 1** (18). The similarity of these loci to *DRB1* can interfere with *DRB1* genotyping. In fact, for many years, most genotyping methods were specifically designed to exclude *DRB3*, *DRB4*, and *DRB5* loci to prevent confounding of *DRB1* genotyping data. Thus, historically, very few studies have reported data for these loci. In addition, the secondary *DRB* loci are far less polymorphic than *DRB1*. Most *DRB1* alleles are found in combination with only one specific secondary allele. DRB1*03:01 represents one exception in that it can commonly be found *in cis* with either DRB3*01:01 or DRB1*02:02 in some populations. The secondary DRB loci have been examined for T1D susceptibility in a limited number of studies with varying results (78–81). Currently, many genotyping products include *DRB3, DRB4*, and *DRB5*; thus, future studies should allow better assessment of their T1D risk.

## 4 Challenges and requirements for determining T1D genetic risk

### 4.1 Diverse population studies

Because of the diversity of HLA alleles and genotype combinations of alleles among populations, limiting the scope of HLA association studies by studying only one or a small number of populations limits the ability to fully understand the full extent of HLA association with T1D. An allele with a strong risk effect could be missed if that allele is present at a frequency too low for proper analysis in the study subjects. Conversely, even modest risk effects can be revealed when an allele is present at a high frequency. In addition, strong LD of some haplotypes creates challenges in the interpretation of data. Effects for one allele in a conserved haplotype may be masked by effects of another allele in that haplotype.

Disease association studies rely on measuring differences between affected and unaffected individuals within a group. A properly designed study must have both case and control groups representing the same genetic background. Given resources to generate sufficient genetic information, this can be determined experimentally, for example, using AIMs; however, in practice, keeping genetic background consistent between cases and controls must be incorporated into selection strategies for study participants. Both consanguinity and population admixture can confound disease association studies; therefore, the selection of study participants is critical. Studies from distinct, carefully selected populations represents a good strategy for understanding HLA-associated T1D risk.

Many studies are performed on the basis of country of origin; however, political boundaries do not necessarily separate race or ethnicity. Conversely, geographic proximity does not necessarily indicate racial or ethnic similarity. One example can be seen in *DRB1* data from Bangladesh and Pakistan (82, 83). Although these two countries both border North India, the HLA associations with T1D are very different, with mild association primarily due to DRB1*04:01 for Bangladesh and strong association, primarily due to DRB1*03:01 for Pakistan. In fact, the pattern of DRB1 association with T1D for Pakistan mush more closely resembles that for Somalia than for Bangladesh (84).

**Figure 3:**
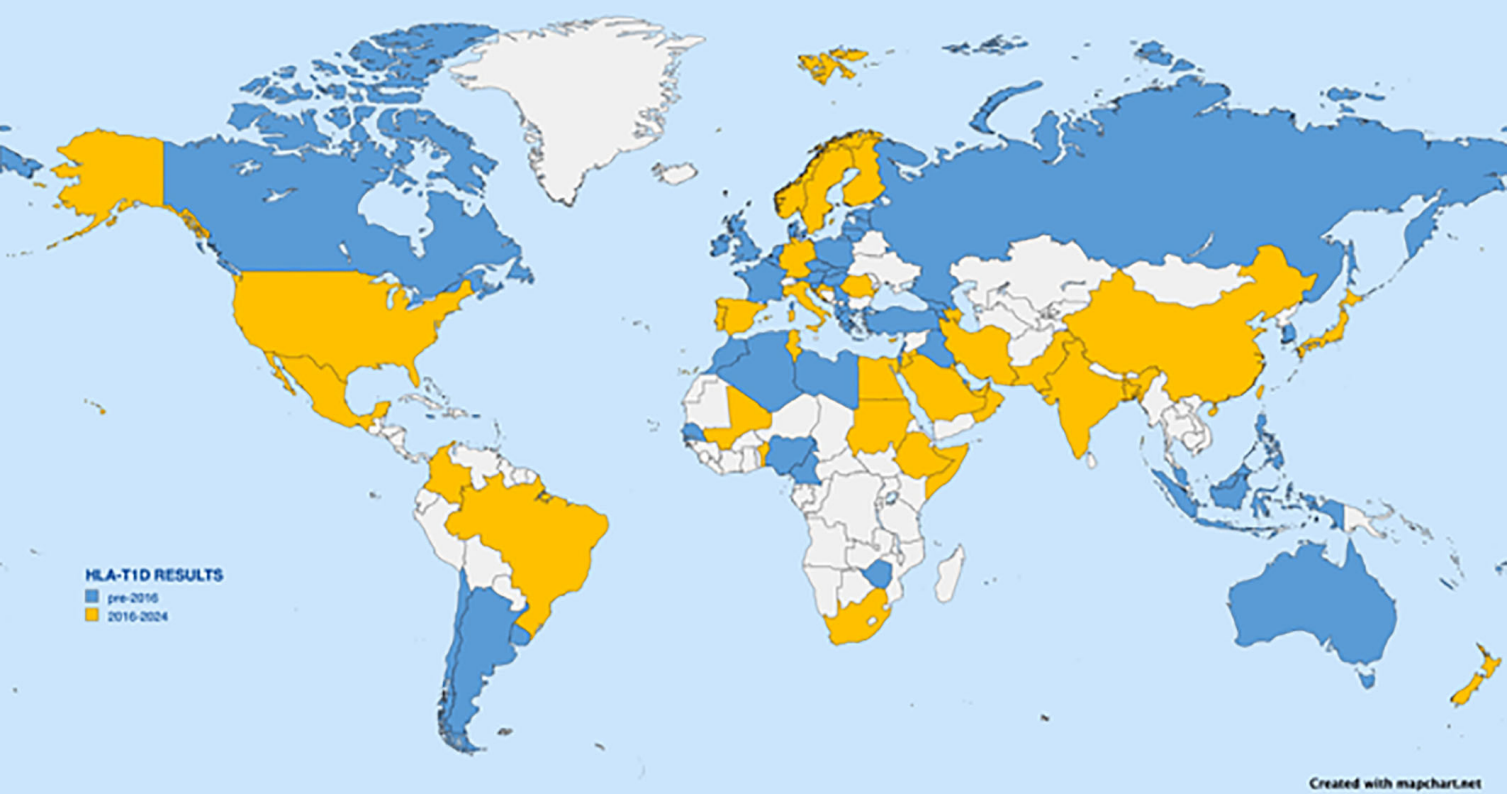
World map depicting countries from which HLA association with T1D have been reported. Any report from a given country, even if from a small region, results in indication of the country. Countries with reports dating from 2016 to June 2024 are shown in yellow. Citations from those countries are listed in **Table 2** and include Albania, Algeria, Argentina, Armenia, Bahrain, Cameroon, Canary Islands (Spain), Chile, Czech Republic, Denmark, Estonia, Fiji, France, Georgia, Georgia, Greece, Hungary, Iraq, Israel, Jamaica, Java (Indonesia), South Korea, La Reunion Island, Latvia, Lebanon, Libya, Lithuania, North Macedonia, Malaysia, Martinique, Morocco, Naura, New Caledonia, Newfoundland (Canada), Nigeria, Philippines, Poland, Puerto Rico, Russia, Russia, Senegal, Serbia, Slovakia, Slovenia, Turkey, Uruguay, Wallis Islands, Zimbabwe. Countries with earlier reported data are shown in blue.

Most early HLA association studies as well as most large-scale studies have been performed on subjects of European descent; however, the number of diverse populations being examined is increasing. A PubMed search using the terms “HLA” and “T1D” returned more than 5,000 publications. **Figure 3** depicts a world map with sources of studies indicated. Although some reports list ancestry or region as the source of subjects, most are reported by country. The data on the map are represented by country; any country from which HLA data have been reported in a T1D study is highlighted, even if the subjects were restricted to a small population or small geographic area. Other than for well-studied areas like the USA, Europe (especially Scandinavia), Japan, China, and India, many reports are from the past 8 years, and most lack replication. The map is not intended to provide the location of each specific population but simply to illustrate the current extent of the world coverage for HLA and T1D association studies. At a glance, the coverage seems quite broad. However, the reported studies vary greatly in the numbers of participants and the geographic area sampled, as well as which HLA loci were tested, which technology was used for genotyping, and what resolution level was reported. **Table 2** lists references seen in PubMed for HLA and T1D studies from 2016 through June of 2024; **Table 2** was used to inform **Figure 3**. Clearly, more data from Africa, South America, and South Asia are needed.

**Table 2:** HLA and T1D reports from 2016 through June of 2024 taken from PubMed.

Country/region/population	Year	First author ^(citation)^
African ancestry	2024	Michalek (166)
Arabian Peninsula	2021	Al Naqbi (167)
Arabs (meta-analysis)	2016	Hamzeh (168)
Azerbaijan	2018	Ahmadov (47)
Bangladesh	2019	Zabeen (82)
Benin	2017	Fagbemi (169)
Brazil	2017	Gomes (170)
Brazil	2023	Gomes (171)
Brazil	2021	Azulay (172)
Brazil	2020	Santos (173)
China	2024	Ding (174)
China	2023	Xia (175)
China	2022	Chen (176)
China	2021	Xia (177)
China	2021	Jiang (178)
China	2020	Ren (179)
China	2017	Yin (180)
China	2016	Sun (151)
Columbia	2019	Gomez-Lopera (181)
Columbia (Antioquia)	2018	Sarrazola (182)
Croatia	2018	Grubik (183)
Cypress	2018	Gerasimou (184)
Egypt	2019	El-Amir (185)
Ethiopia	2020	Balcha (186)
European ancestry	2024	Michalek (166)
Finland	2021	Zhao (187)
Germany, Saxony	2018	Hommel (188)
Hispanic ancestry	2024	Michalek (166)
India	2020	Singh (189)
India	2020	Harrison (117)
India	2019	Kumar (190)
India, North	2024	Kaur (191)
India, North	2021	Chuzho (192)
India, South	2018	Padma-Malini (193)
Iran	2024	Shirizadeh (194)
Italy	2022	Ricci (195)
Japan	2024	Yamada (196)
Japan	2022	Chujo (197)
Japan	2021	Katahira (198)
Jordan	2020	Khdair (199)
Kuwait	2023	Dashti (108)
Kuwait	2023	Haider (200)
Kuwait	2019	Jahromi (201)
Kuwait (Arabs)	2018	Haider (202)
Madiera Island, Portugal	2017	Spinola (203)
Mali	2024	Noble (66)
Mexico	2016	Gomez-Diaz (204)
New Zealand	2022	Willis (205)
Norway	2024	Stordal (206)
Oman	2023	Al-Balushi (207)
Pakistan	2019	Fawwad (83)
Portugal	2023	Caramalho (208)
Qatar	2021	Haris (209, 210)
Romania	2024	Arhire (211)
Sardinia	2017	Incani (212)
Sardinia	2024	Schirru (213)
Saudi Arabia	2020	Eltayeb-Elsheikh (214)
Somalia	2020	Ali (215)
South Africa	2023	Gandini (216)
Spain	2019	Urrutia (217)
Sub-Saharan Africa	2023	Katte (218)
Sudan	2022	Ibaid (219)
Sudan	2021	Ibrahim (220)
Sweden	2021	Alshiekh (78, 221)
Taiwan	2023	Liao (222)
Taiwan	2018	Tung (223)
Tunisia (Arabs)	2019	Hajjej (224)
UAE	2022	Al Yafei (225)
UAE	2021	Tay (226)

Characterization of patients tested is most often reported by country of origin, although some reports use region or ancestry. Reports have wide variation in cohort sizes and in genotyping methodology and resolution. Countries listed in the table correlate to those shown in **Figure 3**.

### 4.2 Good clinical characterization of subjects

An additional confounding factor for T1D studies is the clinical heterogeneity of the disease itself. Traditionally, diabetes has been considered a disease with two categories: type 1, previously referred to as “juvenile” or “insulin-dependent,” and type 2, previously called “adult-onset” or “non-insulin-dependent.” However, many other types of diabetes are now recognized (85). A recent addition to the field is the observation of diabetes as an adverse effect of immune checkpoint inhibitor therapy for cancer (86). Diseases in the category “T1D” are not clinically homogeneous. Presentation of T1D varies among patients from somewhat mild, with minimal insulin requirements, to fulminant, with rapid beta cell loss. Different forms of T1D, termed “endotypes,” are currently being reported, with a recognized need for better global epidemiology (87–89). Different clinical presentation and manifestation of disease may have different HLA associations. Combining subjects with different disease phenotypes could result in inability to recognize some HLA effects.

Yet another variable for T1D involves the antigen presented by the HLA molecules. The search for “the diabetes antigen” has been ongoing for decades. That more than one diabetes antigen exists has become well established, which helps explain why more than just one HLA allele is associated with T1D. Diabetes antigens include peptides formed from proteins recognized by the autoantibodies commonly seen in T1D patients, insulin (IAA), glutamic acid decarboxylase 65 (GAD65) Islet antigen 2 (IA-2), and Zinc transporter 8 (ZnT8). The specific autoantibodies found in T1D patients differ among patients both in the timing of their appearance (IAA tend to appear at earlier age) and the HLA haplotype(s) of the patients in whom they are found (IAA are commonly found in patients who carry DR4 haplotypes, while GADA are more commonly seen in patients with DR3) (90). The discovery that certain antigenic peptides, such as insulin B:9-23, can bind HLA in different registers, with different immunologic consequences, further complicates the immunology of T1D (91, 92). The recent discovery of hybrid insulin peptides (HIPs) adds even more complexity to the diabetes antigen repertoire (93–106). HIPs are created by the fusion of peptide fragments from more than one protein, for example, insulin and chromogranin A. Thus, the number of potential peptide antigens for T1D is much greater than what could be produced from the individual, recognized T1D associated antigens, further complicating the immunologic landscape of the disease.

### 4.3 In-depth analysis of HLA features, rather than alleles

**Figure 4:**
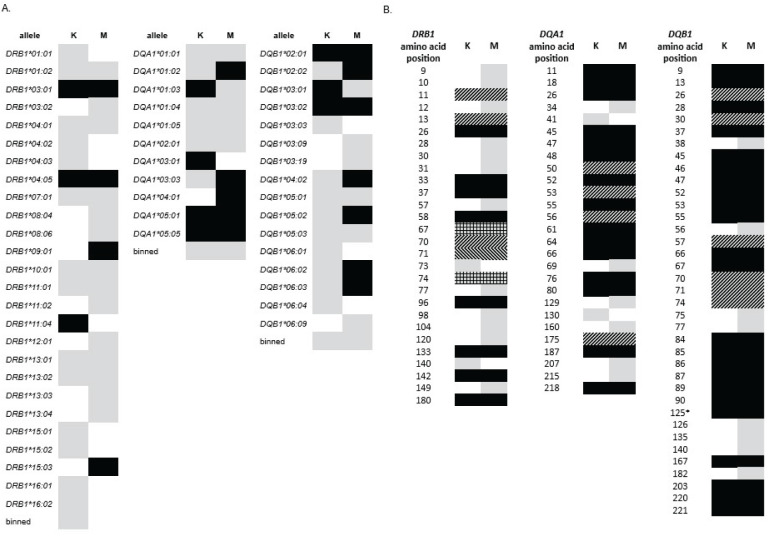
Comparison of data from *HLA-DRB1*, *-DQA1*, and *-DQB1* association with T1D in Kuwait (K) and Mali (M) (108). **(A)** Allele level data. Gray: allele was present in the population at sufficiency frequency for association analysis. Black: allele was significantly associated with disease. “Binned” category contains all alleles that were present but at frequencies too low for individual association analysis. **(B)** Amino Acid position level data. Gray: positions with at least one amino acid residue that shows T1D association not seen in data for the other country. Black: positions in which the same amino acid residues with consistent directions of effect (e.g., predisposing or protective) are seen in both countries. With one exception, each of these positions represents two amino acids, one predisposing and one protective. *Position 125 of DQB1 has only one T1D-associated amino acid residue for each country, but the amino acid and effect direction are consistent. Diagonal lines: positions for which one country shows a greater number of associated amino acids than the other; in all cases, effect direction is consistent for amino acids that appear in both. All but one of these positions shows more associated amino acids for Mali that Kuwait. Only one position, *DRB1* position 71, shows more associated amino acids in Kuwait than in Mali. Lines are shown in the opposite diagonal direction to indicate this difference. Crosshatch: positions for which the two countries share one significant amino acid association, but each has an additional, unique association. Notably, no individual amino acid had an effect that differed in direction between the two countries (i.e., predisposing in one dataset but protective in the other).

HLA antigens for any given locus are structurally similar with small variations due to the amino acids encoded at a given position. Perhaps the most well-known T1D risk association attributed to a specific amino acid residue in an HLA allele is the effect of position 57 in the *DQB1* locus identified in the 1980s (section 3.4) (49, 50). In a much more recent study, effects of individual amino acids on T1D susceptibility were examined in a large cohort of more than 18,000 subjects using imputation of SNP data from the Immunochip array to call HLA genotypes (107). In that study, position 57 of the *DQB1* locus had the strongest effect on T1D risk, with strong associations also seen for *DRB1* positions 13 and 71. In addition, independent T1D associations were observed for amino acid residues in *HLA-A*, *-B*, *-DRB1*, *-DQA1*, *-DQB1*, and *-DPB1* loci; however, no significant associations were observed for either *HLA-C* or *-DPA1*. Consistent with that observation, a recent study of T1D association with HLA in a Maliian cohort showed overall T1Dassociation for all classical HLA loci except *HLA-C* or *-DPA1 (* 66 *).* Looking at T1D association more closely than at the level of the named allele, for example, at the amino acid level, may provide a broader perspective and generate hypotheses to test among studies of diverse populations. An illustrative example can be seen in two recent studies, one from Kuwait and one from Mali (mentioned above), that reported not only allele level but also amino-acid level analysis of T1D patients and controls for the *DRB1*, *DQA1*, and *DQB1* loci (66, 108). These studies on two different populations, one Middle Eastern and one African Black, were of similar size, and both were performed with high-resolution sequencing and analyzed with the BIGDAWG software package (109). **Figure 4A** schematically describes the data observed for each population at the allele level (for those alleles with sufficient frequency to analyze, i.e., not in the “binned” category). Alleles that demonstrate significant T1D association are shown in black. Although they have some overlap, their distributions in these two populations appear different. **Figure 4B** represents the data at the level of individual amino acid residue encoding positions that appear T1D associated in the *DRB1*, *DQA1*, and *DQB1* loci. These data appear more consistent between the countries, with a large proportion of positions showing consistency between the two data sets. One advantage of looking at this level is that it allows examination of all data, regardless of assigned allele names, obviating the need for binning infrequent alleles. Analysis at the amino acid level is likely to provide additional information about T1D risk that could be overlooked at the allele level and may provide a means to pool data from different populations of varying sizes, thereby increasing statistical power.

### 4.4 Strategies to mitigate the high cost of comprehensive HLA genotyping

Given the availability of highly informative DNA-sequencing technology and the knowledge that HLA genes are the most significant contributors to T1D risk, why are full, comprehensive HLA genotyping data available in so few reported studies? Genotyping cost is a major contributing factor. While the evolution of sequencing technology has enabled increasingly comprehensive and accurate genotyping with which to study T1D association, the cost of that genotyping is still quite prohibitive. The probability that full HLA genotyping, or full genome sequencing including HLA data, will soon become inexpensive enough to be applied to the general population, for example, in a newborn screening program, seems quite low. The cost of HLA genotyping has led to the development of techniques for imputation of HLA genotypes from single nucleotide polymorphism (SNP) data. An important consideration for imputed HLA data is that those data, while often correct, represent highly educated guesses and are not experimentally determined. Accuracy of those guesses is dependent on the input data used to train the imputation programs and on the assumptions made in the algorithms. Imputation results for the same data can vary depending on the method used for the analysis (110). However, SNP data are far less costly to obtain than direct HLA genotypes, and can have relatively good predictive power in well-characterized populations, particularly when combined with data for some of the more than ninety other known T1D associated genes (1). This strategy is the basis of development of the genetic risk score (GRS), sometimes referred to as polygenic risk score (PRS) (111–114). A perfect GRS test would allow the prediction of T1D as well as its differential diagnosis from T2D and other forms of diabetes. In individuals of European descent, the GRS performs well to predict T1D in the highest-risk individuals; however, the GRS is reported to perform less well as a diagnostic tool for other races and ethnicities (115–118). Ethnic and racial disparities for GRS or PRS is a general problem and not limited to T1D. Modifications of original GRS panels have improved performance across multiple races and ethnicities (119, 120). Recent reports still vary in conclusions about the utility of current tests, with one report suggesting that the GRS diagnostics are ready for widespread use in the United States while another finds the GRS of only modest predictive power in Australia (121, 122). Undoubtedly, continued refinement of the GRS will create a more universally applicable diagnostic test, but refinement is dependent on overcoming a second hurdle in the quest to fully understand the basis of HLA-associated T1D risk.

Frequencies of HLA alleles differ among ethnicities, as do the haplotype combinations of alleles see section 4.1). Achieving the highest predictive value for a genetic diagnostic test requires knowledge of HLA risk in all populations. Population-specific tests are one way to increase predictive power. However, not all individuals have a single ethnic origin. The United States is sometimes referred to as a “melting pot” with respect to ethnicity and race; consequently, many ethnicities and races are represented in the U.S. population, and many individuals are part of more than one group. Because of the low cost of SNP testing and the ability to multiplex large numbers of tests, an alternate means of increasing predictive value would be to add SNPs for known HLA-associated alleles from all populations into a single test. These SNPs would need to be determined from and vetted in unique populations, underscoring the value of continuing to study classical HLA in even small populations with low T1D prevalence.

## 5 Future directions in T1D risk assessment

### 5.1 HLA expression

Initial studies of HLA association with T1D reported data from serologic typing. The assays were based on using antibodies with known specificities, taken from multi-parous women and standardized among HLA typing laboratories. To see a positive typing result, a particular HLA was required to be present. The advent of DNA-based typing technology meant that measurement of the actual antigen was not required; the presence of a given HLA was inferred from the presence of the gene(s) encoding it. Since then, almost every HLA disease association study has been reported with DNA-based data. As new alleles were discovered, only some inferences about expression levels could be made. If an allele changed an encoded amino acid to a stop codon, it received the designation “N,” meaning no expression of the allele was expected. Very few studies have been reported on the actual expression level of HLA on the cell surface. With six classical HLA antigen types, multiple cell types on which they might be expressed, and the extreme number of alleles for each locus, expression studies are much more cumbersome and expensive to perform than are genotyping assays. Collecting cells from patients for functional studies is much more difficult than collecting DNA to genotype.

DNA-based HLA genotyping determines which alleles are present in an individual sample but, except in rare case (e.g., alleles containing a stop codon), provides no information about differences among alleles at either the transcription or translation level of expression. A limited number of studies have been performed to date attempting to unravel the role of cell-surface expression levels on disease risk. In one such study, a cell surface expression assay was engineered to determine amounts of cell-surface expression as a proxy for the stability of the DQ heterodimeric antigen (54). The authors concluded that T1D association for DQ antigens is inversely correlated with the level of cell-surface protein density, including for heterodimeric proteins encoded *in trans*, such as that formed by the products of DQA1*05:01 and DQB1*03:02 alleles present in a typical high-risk European DR3/DR4 genotype (**Figure 2A**) (53, 54). Given the extent of polymorphism of HLA alleles, testing of every observed HLA antigen is clearly not feasible, but expression testing of select antigens of interest, particularly those HLA class II antigens encoded *in trans*, should be possible.

Now that HLA genotyping technology has improved to the point that whole genes, and even the entire HLA region, can be sequenced with relative ease, the possibility of inferring expression from sequence data has increased. One example of the utility of HLA expression data can be found in the field of transplantation. A SNP in the 3’ untranslated region of DPB1, rs9277534, was found to correlate with low versus high expression levels of DPB1 expression, and high expression of DP is known to be associated with graft versus host disease (123). More recently, LD of that SNP with DPB1 exon 2 sequence was shown to be adequate for prediction of the SNP without direct SNP testing, thereby aiding in transplant donor selection (124). The field of HLA expression and its relationship with disease risk is still in early stages; however, a haplotype of three SNPs in a short region of exon 1 of the DRA gene was shown to be associated with T1D in individuals homozygous for the DRB1*03:01~DQA1*05:01~DQB1*02:01 haplotype (125). Based on eQTL evidence, one proposed mechanism for the effect of this “tri-SNP” haplotype is that it changes, directly or indirectly, expression levels of HLA class II loci. Now that sequencing an entire HLA locus, including untranslated sequences, is becoming routine, non-coding sequences can lead to hypotheses that can be tested in direct functional analyses.

### 5.2 HLA in innate immunity

Studies of T1D to date have focused mainly on the classic role of HLA in presenting peptides to the adaptive immune system. Clearly, HLA plays a major role in initiating the immune response in T1D (class II) and in driving progression of disease via β cell destruction (class I). However, individual allele association studies have not explained all of the risk conferred by the HLA region. The innate immune system also appears to play a role in T1D pathogenesis. Many reports have implicated viruses, inflammation, and interferons as predecessors of disease onset (126–129). Natural killer (NK) cells are key players in the innate immune system and express many activating and inhibitory receptors on their surface, including the widely studied killer-cell inhibitory receptors (KIR) (130, 131). KIR can be either inflammatory or stimulatory depending upon the receptor structure. Loci encoding KIR are found in the leukocyte receptor complex (LRC) at chromosomal position 19q3.4. Like HLA, KIR genes are polymorphic; unlike HLA, much of the polymorphism is attributable to the presence or absence of given genes and their variegated expression, that is, finding a KIR-encoding locus in an individual does not necessarily indicate that its product will be expressed on the cell surface. HLA class I molecules act as ligands for KIR (132). Over the past 20 years, a limited number of studies in several populations have reported attempts to elucidate an association for KIR with T1D (133–156). Not surprisingly, results have been largely inconsistent. A KIR effect would stem from the interaction of a particular KIR with its cognate ligand, requiring that both receptor and ligand be expressed in the same individual, confounding analysis of either molecule alone. Awareness of the extent of KIR polymorphism is in early stages; KIR genotyping studies have evolved from simple “presence or absence” genotyping and copy number variation to genotyping of a growing number of alleles for each locus. Thus, examination of effects of interaction of products of multiple highly polymorphic loci is likely to require extremely large data sets.

LILR genes are also located in the LRC and are largely conserved among primates (157, 158). They have structural similarity to KIR and, like KIR, function to maintain immune homeostasis. LILR are primarily expressed in cells of the myeloid lineage, and their ligands include class I HLA. LILR are reported to be important in infection, transplantation, cancer, and autoimmune diseases, including systemic lupus erythematosus (SLE), ankylosing spondylitis, Crohn’s disease, rheumatoid arthritis (RA), and multiple sclerosis (MS) (157, 159–161). T1D is noticeably absent from this list, and a PubMed search for T1D and LILR returns no results. As sample sets increase and analysis strategies evolve, LILR will undoubtedly be examined for T1D association, perhaps focusing on patients who do not have traditional high-risk HLA alleles.

HLA-C association with T1D is weaker than for HLA-A and HLA-B and is inconsistent among studies. The lack of consistency in the results of HLA-C association analyses with T1D may reflect that the locus is not associated with T1D, and any individual observations of disease association may be spurious or may be attributable to LD with other loci that are T1D associated. An alternate explanation may be that HLA-C influences T1D susceptibility through a mechanism involving the innate immune system, where HLA-C antigens serve as ligands for KIR. A recent study in subjects from Mali demonstrated no locus association with T1D for HLA-C at the allele level; however, analysis at the amino acid level revealed polymorphic amino acid positions that appear to be associated with T1D. One of these was position 309, which is in the transmembrane region and might not be expected to affect T1D risk. However, in a series of transfection-based experiments, the presence of a cysteine at position 309 of HLA-C was shown to inhibit the cytotoxicity of NK cells, suggesting a role for HLA-C in innate immunity (162). The mechanism of such an effect, which might include influencing folding or stability of the protein or its motion in the membrane, remains to be determined.

Association of genes encoding the HLA class II DP antigen also tends to vary widely among studies and among populations (see section 3.5). Recent reports demonstrate that, in addition to classical antigen presenting functions, a subset of HLA-DP molecules can serve as ligands for NKp44, a receptor found on NK cells that can activate them upon ligand binding (16, 163). The interaction of DPB1 with NKp44 has been reported as a risk factor for both primary sclerosing cholangitis, an immune-mediated liver disease, and ulcerative colitis, a form of inflammatory bowel disease (164, 165). Given its apparent role in other immune-mediated diseases, binding of NKp44 to DPB represents a reasonable hypothesis to test for T1D association as well.

## 6 Discussion

More than 50 years have elapsed since the first reports of HLA association with T1D. Those original positive associations of A8 and B15 (now recognized as representing LD with class II DR3 and DR4 haplotypes), negative association of Dw2 (now recognized as DRB1*15 haplotypes), and particularly strong positive association for the Dw3/Dw4 heterozygous genotype, remain valid. They represent, in many cases, the primary contribution of HLA to T1D risk. Does that mean that 50 years of research and more than 5,000 studies were wasted? Not really. Many lessons have been learned from those studies, including (1) HLA-associated alleles should not be considered in isolation but should be analyzed in haplotypic and genotypic context as well; (2) genotyping should be performed minimally at two field resolution, since alleles within a serogroup can have opposite effects on T1D risk; (3) T1D protection is dominant, suggesting that the mechanisms for susceptibility and protection are different; (4) T1D-associated HLA risk can be quite different in different populations, even when they are geographically proximal, due to differences in population allele frequencies and haplotypic combinations. Analysis at the allele level has not yet been sufficient to fully understand how HLA-associated T1D risk works. Full elucidation may require different and more detailed analyses, for example, of gene features and individual amino acid positions, may require very large sample sets, and may require sophisticated techniques to combine old and new data as well as account for population stratification. Still, those original associations with DR3, DR4, DR2 (protection), and the DR3/DR4 genotype remain.

The availability of highly sophisticated genotyping techniques has increased the ability to see exactly what is in the genome of an individual but decreased the ability to determine whether or not the encoded antigens are actually expressed on the surface of the cell. What drives the expression of an allele, on which cells it is expressed, and at what level is it expressed are questions that are just beginning to be addressed. The full extent of the peptide repertoire, both native and hybrid, is currently under investigation and should provide additional clues to the many possible pathways to autoimmunity in patients, perhaps leading to more individualized interventions. Continued refinement of predictive tools, like the GRS, should help identify future patients before overt disease to aid in the efficacy of current (teplizumab) and future intervention strategies.

Adaptive immunity provided by the classical HLA is undoubtedly the major basis for HLA-associated T1D susceptibility; however, the interaction of HLA with the innate immune system represents an intriguing and exciting path to study going forward. Given the solid base of understanding to date and the tools available for continuing study, a thorough and complete understanding of the complexity of HLA-associated T1D risk is unlikely to take another 50 years.
